# Incendiary Leptin

**DOI:** 10.3390/nu12020472

**Published:** 2020-02-13

**Authors:** Patricia Seoane-Collazo, Noelia Martínez-Sánchez, Edward Milbank, Cristina Contreras

**Affiliations:** 1 International Institute for Integrative Sleep Medicine (WPI-IIIS), University of Tsukuba, Tsukuba, Ibaraki 305-8575, Japan; 2 CIMUS, University of Santiago de Compostela-Instituto de Investigación Sanitaria, 15782 Santiago de Compostela, Spain; ed.milbank@usc.es; 3 CIBER Fisiopatología de la Obesidad y Nutrición (CIBERobn), 15706 Santiago de Compostela, Spain; 4 Department of Physiology, Anatomy and Genetics, University of Oxford, Oxford OX1 3PT, UK; 5 Department of Physiology, Pharmacy School, Complutense University of Madrid, 28040 Madrid, Spain

**Keywords:** leptin, thermogenesis, obesity, hypothalamus, brown adipose tissue, browning, white adipose tissue

## Abstract

Leptin is a hormone released by adipose tissue that plays a key role in the control of energy homeostasis through its binding to leptin receptors (LepR), mainly expressed in the hypothalamus. Most scientific evidence points to leptin’s satiating effect being due to its dual capacity to promote the expression of anorexigenic neuropeptides and to reduce orexigenic expression in the hypothalamus. However, it has also been demonstrated that leptin can stimulate (i) thermogenesis in brown adipose tissue (BAT) and (ii) the browning of white adipose tissue (WAT). Since the demonstration of the importance of BAT in humans 10 years ago, its study has aroused great interest, mainly in the improvement of obesity-associated metabolic disorders through the induction of thermogenesis. Consequently, several strategies targeting BAT activation (mainly in rodent models) have demonstrated great potential to improve hyperlipidemias, hepatic steatosis, insulin resistance and weight gain, leading to an overall healthier metabolic profile. Here, we review the potential therapeutic ability of leptin to correct obesity and other metabolic disorders, not only through its satiating effect, but by also utilizing its thermogenic properties.

## 1. The Obesity Pandemic

The World Health Organization defines being obese and overweight as an abnormal or excessive fat accumulation that may impair health [1]. The risk increases when fat accumulation overtakes adipose tissue storage capacity, leading to an ectopic accumulation in other tissues or organs [2,3,4,5,6]—therefore, the regulation and maintenance of energy homeostasis are essential for organism survival. Since energy balance is influenced by a myriad of factors (including genetic, hormonal and nutritional), compounded by environmental and psychosocial variables, the regulation of energy homeostasis is complex. In general terms, the variation in body mass is the result of an alteration of the balance between energy intake and energy expenditure (EE), which are dynamically influencing each other [7] in such a way that individuals with a balanced body mass are protected against an excessive increase or decrease in their fat deposits by compensatory changes in EE [8,9,10].

Obesity is associated with a decrease in life expectancy of between 5 and 20 years and can engender many disabilities depending on the severity of the condition and associated comorbidities [11,12]. These include, among others, hypertension, dyslipidemia, hypertriglyceridemia, insulin resistance and inflammation; cumulatively resulting in a reduced quality of life and social disadvantages [12]. The simultaneous occurrence of several of these factors is known as “metabolic syndrome” and is an important risk factor in the development of diabetes, cardiovascular diseases and/or different types of cancer. The combination of hypercaloric overnutrition, sedentary lifestyle and the evolutionary maintenance of energy-conserving genes [13] has led to an increase of the incidence of obesity in a large portion of the population, independently of the country’s per capita income or development level [14]. Considering the substantial complications of obesity, healthcare spending represents a large budget burden in the developed countries. Due to this, a huge push has been made to identify the underlying molecular mechanisms controlling and regulating energy homeostasis.

## 2. Thermogenesis

Dietary interventions to reduce body weight usually fail due to compensatory changes in EE. In light of this, the role of thermogenesis (i.e., heat production not related to physical activity [15]) in the modulation of EE has been widely investigated and studied. Total EE is the sum of several components: basal metabolic rate (BMR) (approximately 80%), physical activity (10%) and thermogenesis (10%). Of particular note, in active individuals, the combination of both activity and thermogenesis accounts for nearly half (44%) of the total average EE [15,16]. Thermogenesis is a fundamental process used by homeothermic organisms to maintain their body temperature, with the main organ involved in thermogenic regulation being brown adipose tissue (BAT), a specialized adipose tissue mainly involved in heat production, a regulatory process known as “non shivering thermogenesis” (NST) [17]. BAT is especially important in small mammals, as it permits the generation of heat independently of shivering thermogenesis in which heat is produced by involuntary muscle contractions [17,18].

At a histological level adipocytes located in BAT differ from those found in white adipose tissue (WAT). BAT adipocytes possess a polygonal shape with multilocular lipid droplets surrounded by numerous mitochondria [19], containing iron-pigmented cytochromes largely responsible for the brown colour of BAT. Inside the mitochondria, the energy resulting from the movement of electrons through the respiratory chain is classically used by ATP synthase to generate ATP from ADP [20,21,22]; however, this energy-producing process can be modified by uncoupling protein 1 (UCP1). UCP1 provides an alternative way to return protons to the mitochondrial matrix, bypassing ATP synthase and producing heat via thermogenesis [17,23,24].

The adipocytes found in WAT contain few mitochondria and a single large droplet of lipids and are principally involved in fat storage; however, adipocytes displaying similar characteristics to brown adipocytes can be observed in WAT depots by a process called “*beiging*” or “*browning*” of WAT [25]. Apart from their morphological differences, brown and white adipocytes are also originating from different precursor cells [26,27,28,29,30]. While brown adipocytes differentiate from myogenic factor 5 (Myf5) expressing precursor cells (as muscle cells), white adipocytes originate from a distinct Myf5^-/-^ cell lineage [18,31,32]. Interestingly, while possessing similar thermogenic capacities to brown adipocytes, beige adipocytes have the same cellular origin as white adipocytes, differentiating from Myf5^-/-^ precursors [33,34,35]. It should be noted, however, that the in vivo thermogenic capacity of beige/brite (brown-in-white) cells is greatly reduced compared to brown adipocytes [36,37,38,39].

### 2.1. Activation of BAT Thermogenesis

**Figure 1:**
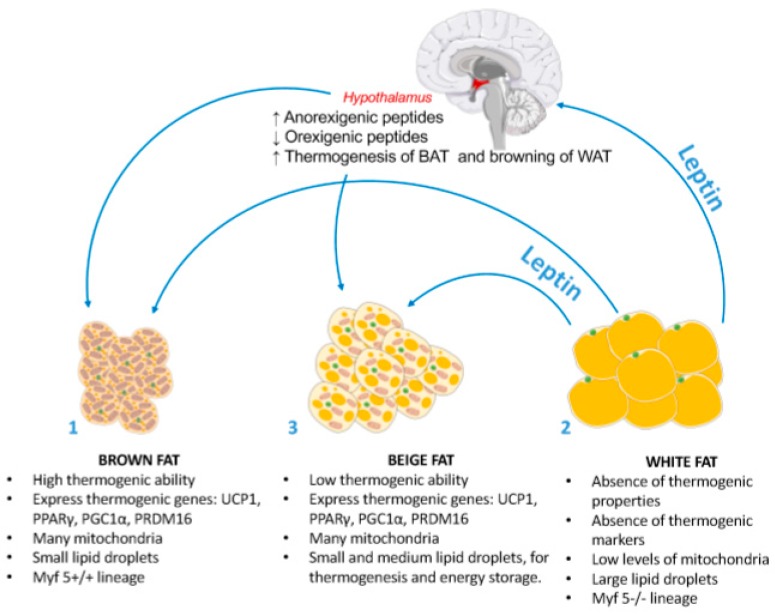
Thermogenic actions of brown, beige and white fat. (1) Brown adipose tissue (BAT) is responsible for heat production to maintain cell homeostasis through a process called thermogenesis. The anatomical features of brown adipocytes are adapted for thermogenesis: many mitochondria using the surrounding multi-locular lipid droplets as fuel to dissipate energy and produce heat. Furthermore, brown adipocytes express several thermogenic markers such as UCP1, PPARγ, PGC1α and PRDM16. Finally, brown adipocytes originate from myogenic (Myf5^+/+^) lineage like myocytes. (2) On the other hand, white adipose tissue (WAT) is responsible for energy storage. In white adipocytes, fat accumulates in large lipid droplets (responsible of white adipocytes big diameter) that occupy the entire cytoplasm. White adipocytes do not carry any thermogenic functions nor thermogenic markers. (3) Beige fat has intermediate anatomical and functional characteristics between white and brown adipocytes. Thus, beige adipocytes emerge in white fat depots bearing thermogenic functions and expressing some thermogenic markers (at a lower proportion than BAT). Beige adipocytes can also store fat. Leptin is released by WAT and exerts its actions at central and peripheral levels. At a hypothalamic level, leptin favours anorexic effects through the overexpression of anorexigenic peptides and down-regulation orexigenic ones. Furthermore, leptin exerts its thermogenic actions in 2 different manners: (i) direct interactions with brown and beige adipocytes and (ii) through hypothalamic actions. UCP1, uncoupling protein 1; PPARγ, peroxisome proliferator-activated receptor γ; PGC1α, proliferator-activated receptor-gamma coactivator 1α; PRDM16, PR domain containing 16.

Brown adipocytes are richly innervated by sympathetic nerve efferent fibres [40,41,42,43,44]. The sympathetic nervous system (SNS) is essential for the activation of thermogenesis that can be regulated both at central and peripheral levels [45,46,47,48,49,50]. The increase of sympathetic tone induces a release of norepinephrine at nerve terminals, that binds and activates β adrenergic receptors (classically β3 adrenergic receptor, β3-AR) localized to the membrane of brown adipocytes. β3-ARs are coupled to G proteins; thus, once stimulated, adenylate cyclase (AC) is activated, triggering an intracellular increase in cyclic adenosine monophosphate (cAMP), which in turn activates protein kinase A (PKA), inducing thermogenesis and subsequently the activation of p38-mitogen-activated protein kinase (MAPK) [17,35,51] (Figure 1).

**Figure 2:**
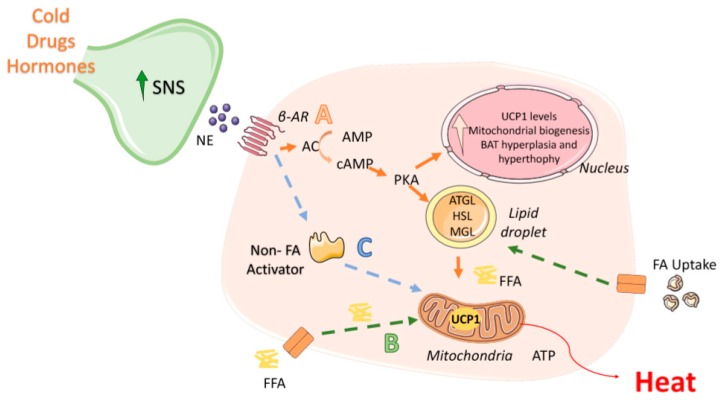
Brown adipose tissue activation. Currently, 3 models of brown adipose tissue (BAT) activation have been proposed. (**A**) (*Orange pathway*) In the classical model, β adrenergic receptors (β-AR) in brown adipocytes are stimulated by sympathetic nervous system (SNS) induced-release of norepinephrine (NE). These G-protein coupled receptors then activate adenylate cyclase (AC), inducing an increase in cAMP, which in turn activates protein kinase A (PKA). PKA acute effect increase lipolysis by the activation of adipocyte triglyceride lipase (ATGL), hormone-sensitive lipase (HSL) and monoacylglycerol lipase (MGL), which hydrolyse the triacylglycerides to release free fatty acids (FFA) that will enter the mitochondria and will eventually be used for heat production by uncoupling protein 1 (UCP1) in the electron transport chain. The chronic effect of PKA activation increases the expression of thermogenic related genes. (B) (*Green pathway*) A second model has been proposed in which thermogenesis is possible even in the absence of ATGL or associated enzymes, mainly by BAT capturing FFA released by white adipose tissue (WAT) and using them as activators and substrates of UCP1. (**C**) (*Blue pathway*) A third model includes the stimulation of brown adipocytes by NE, where a “non-fatty acid” activator activates UCP1 allowing the use of external FFA for the thermogenic process.

Dependent on PKA activation mechanisms as well as mediating pathways, 2 models of BAT responses have been proposed: acute and chronic. During the acute effect, PKA increases lipolysis through the activation of (i) adipocyte triglyceride lipase (ATGL), (ii) hormone-sensitive lipase (HSL) and (iii) monoacylglycerol lipase (MGL), which hydrolyse the triacylglicerides (TAG) to release free non-esterified cytosolic fatty acids (NEFA). Subsequently, NEFAs-CoA are transported into the mitochondria by carnitine palmitoyltransferase 1a (CPT1a) before being oxidized, leading to the subsequent formation of NADH and FADH, which are in turn oxidized in the electron transport chain [35,52,53,54,55]. Recent studies have called into question whether this model could fully explain the mechanism supporting thermogenic activation. In the standard model, UCP1 is inactive being bound to ATP and ADP (or GDP), with this inhibition is reversed by the release of NEFAs from the lipid droplet through ATGL lipase activity [56]. Nevertheless, new evidence has shown that even in the absence of ATGL (or the subsequent ATGL-induced enzymes), thermogenesis remains inducible through BAT capturing NEFAs released by WAT [57,58], which are used as substrates and activators of UCP1. The overriding concern with this model is that the acute role of the SNS is lost/unaccounted for. Therefore, another model has been proposed, in which a norepinephrine-dependent “non-fatty acid” mechanism is required to activate UCP1, allowing its uncoupling and the use of external NEFAs for thermogenesis [56]. Aside from acute effects, the prolonged stimulation of BAT induces a chronic activation of PKA leading to an increase in UCP1 protein levels, mitochondrial biogenesis and BAT hyperplasia and hypertrophy [17,35,56,59] (Figure 2).

### 2.2. Thermogenesis in Humans

Until a few years ago, BAT was considered to be a relevant metabolic tissue only in rodents, hibernating mammals and newborn humans [17,43,59,60]; however, using positron emission tomography-computed tomography (PET-CT), functional BAT was also identified in human adults, and localized in dispersed, yet defined areas of variable sizes, namely: neck, supraclavicular area, perinephric area, intercostal areas and periaortic regions [41,60,61,62]. Its subsequent molecular characterization has shown however, that in the supraclavicular and/or cervical area, its expression profile is more similar to beige/brite adipocytes than to brown adipocytes [25,34,63]. In adults, BAT activity increases as the environmental temperature decreases [62]; accordingly, activated BAT was observed after a period working in a cold environment [64] or after a 2h cold exposure in controlled laboratory conditions [65]. Nonetheless, the magnitude of BAT response to cold exposure is highly variable [62,65] and depends on parameters such as age, sex, body mass index (BMI) or fat mass [65]. For instance, as has observed in aged conditions, the amount and distribution of BAT concomitantly decreases together with an increase in adiposity [66]. Hence a substantial amount of people do not exhibit any signs of BAT activation even after cold exposure [67]. Considering this, several studies have explored the possibility of BAT function recovery by repeated cold exposure showing an increase in glucose uptake by BAT (a hallmark of BAT activity) [68], as well as in BAT volume [69]. Additionally, experiments of sequential monthly acclimation to cold and warm temperatures showed reversible BAT recruitment after cold exposure [70,71].

### 2.3. Thermogenesis, a Therapeutic Treatment for Obesity?

Since the identification of functional BAT in adult humans, numerous studies have been performed to evaluate the therapeutic potential of BAT thermogenesis on metabolic features in humans. A caveat remains, that the relative contribution of BAT to EE is lower in humans than in rodents—while it has been estimated that in rodents thermogenesis could increase the daily EE up to 20% [35,62], maximally activated BAT in humans contributes to approximately 5% of BMR [72].

So far, the main results concerning the therapeutic role of BAT in humans are linked to a reduction in fat mass and to an improvement of glycemic and lipid metabolism. Nevertheless, the evidence gathered by cold exposure approaches have shown none [68] or, in combination with other interventions, a modest [73,74] reduction in body weight and fat mass [75]. Of note, in lean healthy individuals, glucose uptake after BAT activation is increased, improving insulin sensitivity and glucose clearance at a whole body level [76,77,78,79,80]. However, in obese patients, studies have failed to observe any increase in EE, even after recruitment of BAT, probably due to insufficient BAT activation [81]. Whereas in type 2 diabetic patients, cold acclimation was able to increase peripheral insulin sensitivity by augmenting BAT volume and activity [82], suggesting that BAT activation could be an interesting target for diabetes therapy. Interestingly, a possible role of BAT as a regulator of lipid metabolism in humans has also emerged, considering that after cold exposure activated BAT preferentially uses intracellular triglycerides as main substrate for oxidative metabolism instead of plasma glucose or NEFAs [76,83,84,85]. As the number of studies focused on cold exposure in humans has increased, new features of possible effects of BAT activation have emerged. A preliminary study (in which BAT activation was not assessed) performed on hypercholesterolemic patients submitted to prolonged cold exposure showed a reduced body mass associated to an improved total cholesterol and LDL cholesterol, independently of physical activity or changes in food intake [86]. Furthermore, it was described that individuals with cold-activated detectable BAT presented lower plasma cholesterol and LDL cholesterol compared to subjects without detectable one [65]. In any case, further studies are needed to assess if hypercholesterolemia could also be alleviated by BAT activation.

More recently, increasing efforts to target BAT in an obesity-driven context have been made, focusing mainly on the pharmacological activation of thermogenesis instead of temperature interventions [87]. These innovative BAT pharmacological interventions were developed mainly due to the fact that (i) BAT activity is inversely correlated with BMI [60,62] and (ii) because obese subjects are less prone to respond to cold-induced thermogenesis [88], probably due to greater adipose insulation. The initial pharmacological candidates were β3-AR agonists; however, almost all have been discarded following clinical trials due to the lack of effectiveness, toxicity issues and/or crossover effects with β1- and β2-AR [89]. Nevertheless, studies have found that mirabegron, a selective β3-AR agonist approved for the treatment of overactive bladder, was able to increase BAT activity and WAT lipolysis in healthy male subjects in the same range of efficiency as cold exposure [90]. In agreement with this, a recent clinical trial has found that both mirabegron and cold exposure (even if limited to a small surface skin area) induced an increase of beige adipose tissue markers in human subcutaneous WAT [91]. Of note, this effect was observed in obese insulin-resistant subjects pointing out the possible therapeutic use of mirabegron to treat obesity, although further studies are needed to determine whether these effects on WAT beiging are associated to a better clinical outcome.

Other factors inducing BAT activation have been explored in several studies such as (i) the sympathomimetic compound ephedrine that was able to increase EE (independently of BAT activation) [92], (ii) capsinoids (although providing contradictory results on EE modulation) [93,94,95,96], (iii) bile acids—inducing an increase of BAT activity [97] or (iv) leptin and leptin sensitizers which will be discussed later in this review.

In this therapeutic context, it is important to mention that mutations in the *UCP1* gene could be involved in the pathology of obesity. In this sense, some nucleotide substitutions in the *UCP1* gene were described in obese people suggesting that specific variations of *UCP1* could promote energy storage and contribute to the development of obesity [98]. Other specific *UCP1* polymorphisms, such as rs1800592 and rs3811791, are associated with obesity and abnormal values of high-density lipoprotein (HDL), low-density lipoprotein (LDL) and triglycerides levels [99]. It has also been shown that the UCP2-866 polymorphism was associated with high levels of leptin in an obese or overweight Mexican population [100]. Of note, these studies have failed to find any strong general association between other *UCP1* polymorphisms and obesity, instead relying on specific parameters or obesity levels [99], highlighting the remarkable complexity of the topic.

## 3. Leptin History

Historically WAT has been considered as a passive energy storage organ; however insurmountable evidence has demonstrated that WAT possesses a major endocrine function through the release of several factors into the circulation. In 1987, the first description of adipose tissue being a source of sexual hormones was made [101]. The next discovered factor released by adipose tissue was adipsin, which was markedly decreased in obese rodent models [102]. In 1994, leptin was identified and characterized, cementing the endocrine function of adipose tissue [103]. Nowadays a plethora of hormones and peptides released by adipose tissue have been identified such as: interleukin (IL) 6, tumour necrosis factor-α (TNF α), monocyte chemoattractant protein (MCP) 1, plasminogen activator inhibitor (PAI)-1, resistin, adiponectin, to name a few. Collectively termed adipokines, they can act in autocrine, paracrine or endocrine manners, establishing a complex communication between adipose tissue and other organs, including the brain [104].

Before the identification of leptin, several studies had already suggested the existence of a particular circulating endocrine hormone capable of signalling information to the central nervous system (CNS) about the energy status/requirements [105]. This theory was later confirmed by Friedman and colleagues through the identification of the leptin gene (*Lepob*) [103] that encodes for a 16 kDa polypeptide composed of 167 amino acids, named Leptin from the Greek λεπτός (leptos), meaning “thin”. Subsequently, the leptin receptor (*LepR*: *Lepdb* in mice and *Lepfa* in rats) was described [106,107,108]. Leptin production is regulated by the *Lepob* gene in adipocytes, and therefore by lipid content and adipocyte size [109], meaning that circulating leptin levels directly correlate with adipose tissue mass (elevated numbers of subcutaneous depots compared to visceral ones) and nutritional status [110,111]. It should be noted however, that many other factors can regulate the expression and secretion of leptin.

The conservation of leptin across different species and diverse organs points to the functional relevance of this adipokine [112] and its subsequent involvement in the regulation of many biological systems including: energy homeostasis, endocrine systems, immune function, hematopoiesis, angiogenesis and bone development [104,113,114,115,116,117,118,119,120,121].

In order to understand metabolic pathologies such as obesity or type 2 diabetes, genetically modified rodent models bearing alterations in leptin signalling pathway have been developed throughout the years. Most famously, ob/ob mice completely lack functional leptin due to a single autosomal recessive mutation in *Lepob* [103,122], whilst db/db mice carry a single autosomal recessive mutation in the *LepR* gene resulting in abnormal and nonfunctional LepR. Of note, while db/db mice (lacking LepR) display high levels of leptin, they are resistant to its effect [106,107]. In addition to mice, rat models exhibiting alterations in leptin signalling have also been generated. For instance, Zucker fatty (fa) rats harbour a mutation in the *LepR* gene resulting in nonfunctional receptor [123]. Interestingly, all these aforementioned models develop genetic obesity and enter a prediabetic state, mimicking clinical progressions and are therefore widely used to study these pathologies and leptin signalling pathways.

## 4. Leptin Anorexigenic Effects

The effects of leptin on energy balance are mainly due its food intake suppressing properties and to its ability to induce thermogenesis. Regarding its primary function, leptin is considered to be the main ‘satiety hormone’ [124,125,126,127,128]. Accordingly, when injected in leptin-deficient patients, leptin is able to normalize hyperphagia through a reduction of food intake [129,130] and to decrease hunger without affecting satiety in adults [131].

The major neuronal targets of leptin are located in the hypothalamus, a brain area located under the thalamus mainly involved in the energy balance regulation, that is composed of distinct neuronal populations grouped in hypothalamic nuclei forming the: arcuate (ARC), ventromedial (VMH), paraventricular (PVH), dorsomedial (DMH), nucleus of the hypothalamus and lateral hypothalamic area (LHA) [106,119,132,133,134,135]. Interestingly, it has been demonstrated that the ARC performs a key role in mediating leptin actions [136], with two notable subpopulations of neurons expressing LepR:

(1) Pro-opiomelanocortin (POMC) neurons that express anorexigenic neuropeptides such as POMC and cocaine- and amphetamine-regulated transcript (CART) [137,138], and are stimulated by leptin through the release of α-melanocite-stimulating hormone (α-MSH) [139,140,141] that binds the melanocortin 3 receptors (MC3R) and MC4R. Several genetic variants of *POMC* and *MC4R* genes have been associated to human obesity, suggesting that the central melanocortin system is required for the anorexigenic effect of leptin.
(2) Agouti-related protein (AgRP) neurons [142,143] which express orexigenic neuropeptides such as neuropeptide Y (NPY) and AgRP [144]. Leptin exerts inhibitory effects on both AgRP and NPY neurons activity as well as on the release of the associated AgRP and NPY neuropeptides [142,145], resulting in a potent satiating effect.

## 5. Molecular Mechanisms Mediating Leptin Effect

The leptin signalling pathway is initiated when leptin binds its receptor located in central and peripheral organs. The *LepR* gene encodes for 6 LepR isoforms (LepRa to LepRf) through differential mRNA splicing processes, with a conserved N-terminal intracellular domain. LepRb is the only isoform that has a full-length intracellular domain and is involved in leptin signalling [106,146,147]. The hypothalamus is the main area in which leptin’s anti-obesity effects are mediated [148,149]. How other isoforms of LepR are involved in leptin signalling remains unclear; however, it is likely that they could be implicated in the transportation and clearance of leptin [145,150,151].

**Figure 3:**
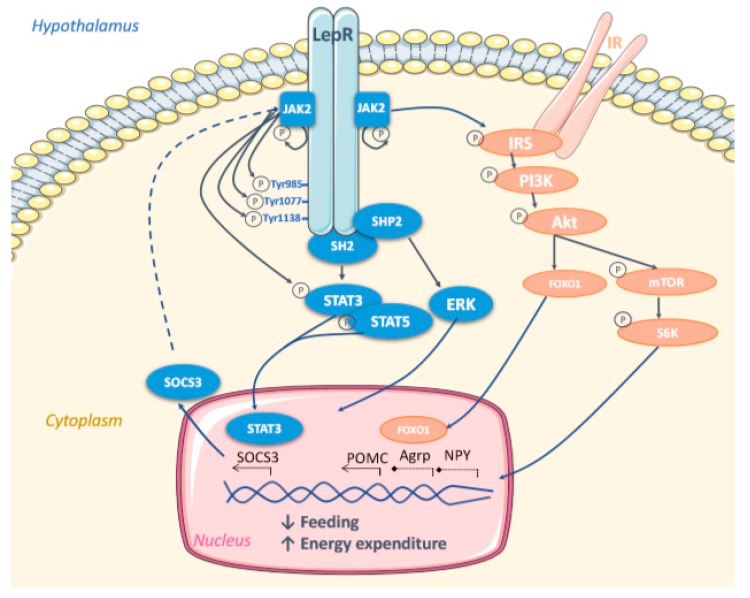
Leptin-induced pathways. The binding of leptin to its receptors (LepR), mainly expressed in the hypothalamus, leads to its dimerization and recruitment and auto-phosphorylation of Janus tyrosine kinase 2 (JAK2). JAK2 subsequently phosphorylates LepR on 3 distinct tyrosine residues (Tyr985, Tyr1077 and Tyr1138), facilitating the binding of Src Homology 2 (SH2) containing molecules. Depending on the phosphorylated tyrosine, different pathways will be activated. Phosphorylation at Tyr985 allows the binding of (i) Suppressor Of Cytokine Signalling 3 (SOCS3) inducing negative feedback to inhibit JAK2 and (ii) SH2 domain protein tyrosine phosphatase 2 (SHP2) to activate Extracellular-signal Regulated Kinase (ERK) pathways. The Tyr1077 permits the phosphorylation and activation of Signal Transducer And Activator of Transcript 5 (STAT5) leading to gene expression changes. Finally, the Tyr1138 allows the phosphorylation of STAT3 altering gene expression and activating SOCS3, reinforcing the negative JAK2 feedback. Furthermore, the leptin pathway is interconnected with the insulin pathway, since JAK2 activation phosphorylates Insulin Receptor (IR) and Substrate (IRS), and subsequently, Phosphatidyl Inositol 3 Kinase (PI3K), protein kinase B (Akt) leading to the activation of Forkhead box protein O1 (FOXO1) and also mammalian Target Of Rapamycin (mTOR)/ribosomal S6 kinase (S6K). Together, all these leptin-activated signalling pathways trigger energy homeostasis gene regulation, mainly consisting in over-transcription of anorexigenic Pro-opiomelanocortin (POMC) and down-transcription of orexigenic Agouti Related Protein (Agrp) and Neuropeptide Y (NPY) neuropeptides, as well as favouring energy expenditure genes, including thermogenic markers.

As LepRb does not contain any intrinsic enzymatic activity, its association to a cytoplasmic tyrosine kinase—Janus tyrosine kinase 2 (JAK2)—is needed to initiate the subsequent leptin-mediated molecular mechanisms. The binding of leptin to dimerized LepRb stimulates JAK2, inducing its activation by auto-phosphorylation allowing the phosphorylation of LepRb on three tyrosine residues—Tyr985, Tyr1077 and Tyr1138. When phosphorylated, these tyrosine residues act as binding sites for Src homology 2 (SH2) domain containing molecules triggering the corresponding downstream signalling pathways (Figure 3).

On one hand, in response to leptin, JAK2 phosphorylates LepR on Tyr1138 triggering the recruitment of the Signal Transducer and Activator of Transcript 3 (STAT3) through its SH2 domain. STAT3 is subsequently phosphorylated by JAK2, resulting in its dimerization and nuclear translocation in order to regulate the expression of STAT3-target genes, including Suppressor Of Cytokine Signaling 3 (SOCS3) [152,153,154,155]. STAT3 acts as a transcription factor essential for feeding regulation [149,156,157,158,159]. Moreover, leptin can activate STAT5 through the phosphorylation of LepRb on its Tyr1077 and Tyr 1138 (partial phosphorylation) residues. Interestingly, both STAT3 and STAT5 deletion have been associated with obesity and hyperphagia states.

On the other hand, phosphorylation of LepR on Tyr 985 provides a binding site for the SH2 domain protein tyrosine phosphatase 2 (SHP2) which regulates Extracellular Signal Regulate Kinase (ERK) pathway, that is known to be associated with both thermogenic and anorectic effects of leptin [160]. Phospho-Tyr985 can also be triggered by SOCS3 which exerts a negative feedback effect suppressing the activation of the LepRb/JAK2 pathways [161]. It is important to include here that a selective mutation in Tyr985 improved leptin signalling in lean mice, highlighting the role of Tyr985 in this negative feedback signalling [162,163].

Finally, the activation of JAK2 following leptin binding to LepRb promotes the phosphorylation of insulin receptor substrate 1 and 2 (IRS1 and 2) inducing the activation of the Phosphatidylinositol 3-Kinase (PI3K)/protein kinase B (Akt) pathway [164,165,166,167,168,169] and of its two corresponding downstream events: (1) phosphorylation of Forkhead box protein O1 (FOXO1) that is translocated from the cytosol to the nucleus, promoting the transcription of POMC and the inhibition of AgRP and NPY transcription, leading to the suppression of food intake [170,171,172]. In this sense, it has been shown that an overexpression of *FoxO1* in the ARC abolishes leptin response increasing feeding and body weight, while its deletion results in an inverted phenotype [170,171,173]; (2) The other downstream event derived from IRS/PI3K/Akt pathway is the activation of the mammalian Target Of Rapamycin (mTOR)/ribosomal S6 Kinase (S6K) [142]. It has been demonstrated that the hypothalamic activation of mTOR complex1 (mTORC1) decreased food intake and body weight in rodent models, while rapamycin, its suppressor, had orexigenic effects [174], suggesting that mTOR plays a crucial role in the leptin-mediated regulation of energy homeostasis.

Besides the classical leptin-dependent signalling pathway, the Calcium Calmodulin-dependent protein Kinase Kinase (CaMKK2)/5′-AMP-activated protein kinase (AMPK)/acetyl-CoA caroboxylase (ACC) pathway has been demonstrated to be involved in leptin receptor signalling [142,175,176]. AMPK is typically activated under decreased intracellular energy levels (i.e., low ATP/ADP ratio) [177]. Thus, while leptin signalling is activated by increased levels of glucose, AMPK is activated when they are decreased. Interestingly, the pharmacological inhibition of AMPK [178] restores the leptin signalling pathway, suggesting that CaMKK2/AMPK/ACC signalling exerts a key role in the modulation of the leptin-dependent pathways.

## 6. Leptin Effects on Thermogenesis and Browning

In addition to its actions regulating food intake, leptin also positively regulates energy expenditure and thermogenesis. Deficiency in leptin or in LepR leads to a decrease in energy expenditure and in core body temperature [179,180]. Indeed, ob/ob mice present (i) a reduced SNS activity, (ii) lower expression of β3-AR [181,182] and (iii) decreased body temperature [126,183,184]. On the other hand, many agents (cold exposure, hormones, β3-AR agonists, dietary factors and exercise among others) have been described to be clear browning inducers [50,185,186,187] while the exact role of leptin in WAT browning still remains unclear. In the following section, the central and peripheral mechanisms of leptin involved in thermogenesis and browning processes will be reviewed.

### 6.1. Thermogenic Effects of Leptin

**Figure 4:**
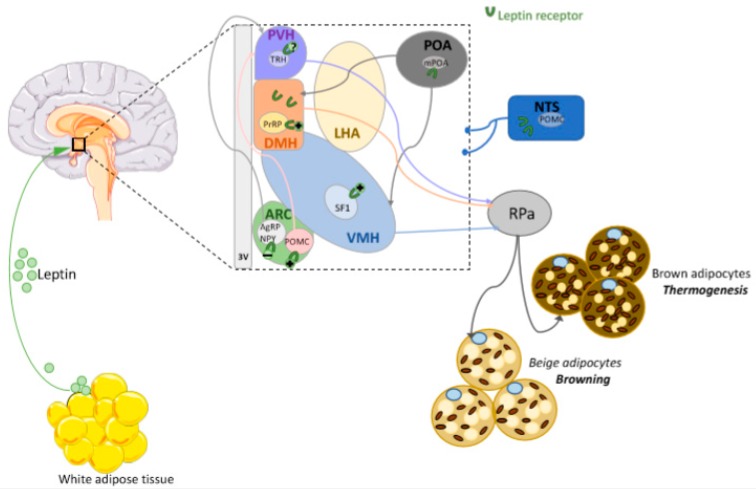
Hypothalamic leptin-sensitive neurons regulating thermogenesis and browning. The leptin receptor (LepR) is highly expressed in several hypothalamic areas implicated in the thermogenic program. The Dorsomedial Hypothalamus (DMH) (high density of LepR), the Preoptic Area (POA) and the Steroidogenic Factor 1 (SF1) expressing neurons of the ventromedial hypothalamus (VMH) have a significant role in the control of the Sympathetic Nervous System (SNS) outflow to the brown adipose tissue (BAT) and the white adipose tissue (WAT) to control thermogenesis and browning respectively. Neurons in these areas are interconnected and can control the sympathetic activity regulating downstream effector neurons in the Raphe Pallidus (RPa). The Arcuate nucleus of the hypothalamus (ARC) also has high expression of LepR: different ARC neuronal populations exert opposing effects on the thermogenesis through the inhibition of Agouti-Related Protein (AgRP)/Neuropeptide Y (NPY) neurons and activation of Pro-opiomelanocortin (POMC) ones. These neurons can act on the Paraventricular nucleus of the hypothalamus (PVH) to decrease or induce thermogenesis respectively. LepR is also expressed in extra-hypothalamic areas, such as POMC neurons in the Nucleus of the Solitary Tract (NTS); these neurons project to different hypothalamic nuclei but their actions are distinct from the POMC expressing neurons of the ARC. Although LepR is expressed in the PVH the role of the PVH in regulating thermogenesis is not completely clear, likely via indirect connections with another nucleus. TRH: thyrotropin-releasing hormone. PrRP: prolactin releasing peptide. mPOA: medial POA.

The role of leptin in BAT thermogenesis stimulation was demonstrated years ago. Of note, leptin exerts its thermogenic action mainly through the activation of the SNS. In rodent models, peripheral or central leptin injections increase sympathetic nerve activity innervating BAT (an effect that depends on the integrity of LepR) [188,189,190]. Moreover, in obesity models (diet-induced-obesity and ob/ob mice), intraperitoneal injection of leptin increases BAT UCP1 mRNA levels and activity without causing significant changes in mice locomotor activity [191,192]. As mentioned above, the hypothalamus has the highest expression of LepR and interestingly, many of the hypothalamic neurons involved in the regulation of the thermogenesis are also leptin sensitive (Figure 4).

Before diving into leptin’s actions and effects on different hypothalamic areas, it is important to mention that some authors have not observed any association between thermogenesis and leptin. Leptin-deficient ob/ob mice have been characterized as thermogenic limited and hypothermic due to atrophied BAT. However, other studies have demonstrated that the BAT of these mice were perfectly functional and that leptin treatment did not increase BAT thermogenesis. Recently, Fischer and colleagues observed that leptin administration in wild type and ob/ob mice did not induce any thermogenic response. Surprisingly, they observed that ob/ob mice displayed a decreased body temperature that was normalized to wild-type levels after leptin treatment [193]. As other authors have obtained similar results, highlighting the idea that leptin may not be considered as a thermogenic but as a pyrexic one [194,195,196,197,198,199], further studies are warranted to clarify leptin’s exact role in regulating thermogenesis.

### 6.2. Leptin Actions in the DMH

Some evidence suggests that many neuronal populations in the DMH are important for the regulation of BAT thermogenesis [200,201,202]. In regards to the main topic of this review, it is important to note that the neurons composing the DMH display a very high density of LepR [203] and that their activity is sufficient to promote BAT thermogenesis, increase energy expenditure and locomotor activity—cumulatively leading to a decrease in body weight [204]. Concordantly, the ablation of LepR in DMH neurons results in weight gain by decreasing EE and locomotor activity [204,205]. Specifically, some authors have shown that after intraperitoneal injection of leptin, DMH phospho-STAT3 expression increased, inducing sympathetic activation and increased BAT activity [192,206]. Accordingly, following intra-DMH leptin injection, BAT temperature also increased and that this effect could be blocked by prior infusion of β3-AR antagonist [192]. Additionally, some authors have shown that the disruption of LepR in the prolactin releasing peptide (PrRP) neurons (a specific DMH neuronal subset associated with thermogenesis), induced a decrease in the thermogenic effects of peripheral leptin [207]. DMH LepR-expressing neurons project their axons to different brain regions including the PVH [203,208] and the Raphe pallidus nucleus (RPa) [209,210], areas that are involved in the sympathetic regulation of crucial physiological parameters such as body temperature, blood pressure or heart rate [203,211]. Moreover, Zhang and co-workers demonstrated that LepR in the DMH mediates the thermoregulatory actions of leptin through the use of a retrograde trans-synaptic tracer [211]. Taken together, these findings support the idea that leptin, through its actions on leptin-sensitive neurons of the DMH, plays a role in the stimulation of the BAT thermogenesis and in the control of body temperature.

### 6.3. Leptin Actions in the Preoptic Area (POA)

The POA is the main region involved in temperature sensing and in the integration of peripheral and central thermal information emanating from the organism. The medial POA (mPOA) detects cold temperature signals and induces a thermogenic response in BAT through hypothalamic outputs [212,213]; in this way, the mPOA plays an important role in sympathetic/thermogenic BAT circuits. More precisely, mPOA neurons project their axons directly to neurons of the DMH to regulate sympathetic BAT inputs and the associated thermoregulatory responses [200,214,215]. As in the DMH, neurons expressing LepR have been found in the mPOA [143,211] and are involved in sympathetic circuits activating BAT and in thermoregulatory leptin actions [216]. Zhang and colleagues [216], using a retrograde transsynaptic tracer pseudorabies virus (PRV), revealed that LepR neurons of the mPOA contributed to the regulation of the sympathetic BAT outputs. These LepR BAT-related neurons project to the DMH/dorsal hypothalamic area (DHA) and to the RPa forming an interconnected circuit in which leptin could act at different levels [163]. Remarkably, mPOA neurons express MC4R and the specific mPOA injection of its agonist, melanotan II (MTII), was shown to increase thermogenesis [217]. As the mPOA modulates excitatory DMH neurons projecting to the RPa [218,219], this effect is ablated by lesioning the DMH [217].

### 6.4. Leptin Actions in the PVH

The PVH plays an important role in the regulation of energy homeostasis and is interconnected with other hypothalamic areas. However, the role of LepRb in the PVH remains controversial as most of the evidence is indirect and unclear [220]. However, some reports suggest that both oxytocin and also thyrotropin-releasing hormone (TRH) neurons located in the posterior pituitary gland and the PVH, respectively, express LepR and could modulate EE [221]. Therefore, it has been suggested that the PVH could act as a final common pathway in response to leptin. For example, DMH neuronal efferents project to parvicellular PVH areas [222,223,224] that directly innervate parasympathetic and sympathetic preganglionic neurons in the medulla and spinal cord [137]. Some results support the idea that a subset of leptin-sensitive cells in the DMH innervate the PVH [208,211], but the functional significance of these projections needs to be clarified with further studies.

### 6.5. Leptin Actions in the VMH

One of the main roles of the VMH is the regulation of thermogenesis through the integration of several peripheral signals to produce a thermogenic response in BAT and WAT. Therefore, numerous peripheral signals relaying the nutritional status (as nutrients and hormones, including leptin) are conveyed to the VMH, in which high concentrations of LepR have been described [132]. The role of leptin in the VMH on thermogenesis modulation is well accepted, indeed microinjections of leptin into the VMH have been shown to increase epinephrine and norepinephrine plasma concentrations [225]. Other studies supporting this VMH leptin-mediated sympathetic activation have shown that leptin infusion into the VMH increases glucose uptake in peripheral tissues [226], as well as blood pressure and renal sympathetic activity [227,228]. Specifically, after leptin microinjections into the VMH, changes in BAT activity were detected with increased BAT glucose uptake. Interestingly, this effect was blocked after specific sympathetic denervation of BAT [226]. The dorsomedial area of the VMH is especially enriched in LepRb and in the specific transcription factor Steroidogenic Factor-1 (SF1) [229,230]. Leptin directly activates SF1 neurons in the VMH inducing modulation of body weight. The use of mice models lacking LepR in SF1 neurons has demonstrated that these neurons mediate, at least in part, the anti-obesity effect of leptin. Specifically, when challenged with high fat diet (HFD), these SF1 LepR^-/-^ mice develop an increase in body weight and fat storage without hyperphagia, which was explained by a defective adaptative thermogenic response accompanied by decreased BAT UCP1 expression [231,232]. Curiously, VMH specific SF1 KO mice display significantly reduced VMH LepR expression [232]. It is important to note that the SF1 neurons are mostly glutamatergic [233,234], exerting output functions involved in the regulation of sympathetic nervous system outflows [235]. Thus, the deletion of these neurons induces a decrease in sympathetic outflow and, as a result, a decrease in thermogenesis. Other studies have shown that LIM domain only 4 (LMO4), a transcription cofactor essential in CNS development, is also involved in central leptin signalling. This cofactor is expressed in specific nuclei of the hypothalamus, including the VMH [236]. Chronic intracerebroventricular (ICV) leptin infusion in mice with neuronal specific ablation of LM04 induced a decrease in thermogenesis and energy expenditure, while the leptin-induced weight and fat loss were less marked [237]. Thus, this study proposed LMO4 as a novel modulator of leptin function in selective hypothalamic areas. Moreover, recently, a link between Cannabinoid type-1 (CB1) receptors expressed in VMH neurons and the metabolic actions of leptin was established. Mice lacking CB1 receptors specifically in SF1 expressing neurons possessed an increase in sympathetic activity and a decrease in adiposity when fed with a standard diet. Conversely, under HFD conditions, these mice developed leptin resistance and increased peripheral adiposity [238], suggesting a diet-dependent role of the SF1-VMH CB1 receptors in energy balance and metabolic responses to leptin. The regulation of leptin activity in neurons also implicates the transcription factor FOXO1 that plays a central role in metabolic homeostasis, with the VMH being a key site for its action [173]. Since FOXO1 is considered to be a negative regulator of leptin (via PI3K/pAKT pathway), its specific ablation in SF1 neurons of the VMH, induces an increase of SNS activity and UCP1 expression, and consequently EE [239]. Recently, new studies were performed in rat models, demonstrating that weight loss in leptin-treated rats only occurs in combination with the simultaneous activation of LepR in the hindbrain and forebrain, with a critical role of the VMH in this interconnected network [240].

### 6.6. Leptin Actions in the ARC

As previously mentioned, the ARC is a hypothalamic nucleus with high levels of expression of the LepRb isoform. The ARC is closely related to the control of leptin-dependent feeding, but is also an important area for the leptin-induced increase in BAT sympathetic outflow. Several studies have demonstrated that the ARC was involved in leptin response, indeed the sympathetic activation of BAT observed after systemic administration of leptin was blunted after specific electrolytic lesions of the ARC [241]. On the other hand, direct injections of leptin into the ARC induced the increase of BAT sympathetic tone and blood pressure [242]. Moreover, the selective deletion of LepRb in the ARC reduced BAT sympathetic nerve responses to leptin [242,243].

More specifically, the two neuronal populations expressing either the orexigenic peptides AgRP/NPY or the anorexigenic peptide POMC, are the principal sites of LepR expression in the ARC and exert opposing effects on metabolism [244]. In addition to its role in the decrease in EE, ARC and NPY neurons are critical for the sympathetic control of BAT function [245]. The connection between NPY and leptin has been long recognized: on one hand, NPY is overexpressed in the hypothalamus of ob/ob mice [246], and its repetitive administration into the CNS was shown to suppress the SNS activity of BAT, decreasing energy expenditure [247,248] and inducing obesity [249]. On the other hand, ob/ob mice treated with leptin displayed lower ARC NPY expression [127,134] and the single or repetitive central administration of leptin decreases NPY mRNA levels in the ARC [250,251,252]. On the basis of these findings, NPY seems to be a strong mediator of leptin thermogenic actions. More precisely, leptin could act centrally to stimulate BAT activity and this effect may be partially mediated by the inhibition of NPY neurons of the ARC [253]. It is also important to mention that NPY neurons project to both the PVH and the VMH and that these neuronal connections are implicated in NPY-mediated thermoregulatory effects [248,254].

The second ARC main neuronal population—POMC expressing neurons—which are part of the melanocortin system, have also been linked to leptin action [141,255]. The activation of LepR in POMC neurons has hypertensive effects, increasing blood pressure and heart rate [256]. Although POMC expressing neurons are also found in the nucleus of the tract solitarius (NTS), leptin does not stimulate them [257], suggesting that leptin can differentially regulate these two POMC neurons populations. In addition, MCR3 and MCR4 are important mediators of leptin action in the neurons of the melanocortin system [258,259]. The co-administration of leptin and SHU9119 (a synthetic antagonist of both MCR3 and MCR4 receptors), attenuated leptin-induced anorexia [260] and completely inhibited leptin-induced BAT increase in UCP1 mRNA levels [225,261]. Furthermore, central administration of the MTII MC4R agonist had opposite effects, i.e., increasing BAT UCP1 mRNA levels by stimulating SNS activity [261]. Interestingly, this effect was blunted following sympathetic denervation [262]. Moreover, peripheral leptin treatment of MC4R-null mice was neither able to induce UCP1 expression in BAT [263] nor the leptin-dependent renal sympatho-excitatory response [264]. After HFD or cold exposure (external factors known to disturb leptin production), mice showed a diminished upregulation of BAT UCP1 [265]. Together, this suggests that leptin signalling in POMC neurons regulates in part the SNS outflow through an MC4R dependent mechanism.

The association between the melanocortin system and metabolic rate remains controversial. In this regard, the injection of MTII was shown to induce hypothermia/hypometabolism (achieved by decreasing BAT thermogenesis) before engendering the opposite effect (i.e., hyperthermia/hypermetabolism) [266]. In the same study, it was observed that this hypometabolic effect was preserved in MCR 1-, 3-, 4- and 5-knockout mice, indicating that these receptors did not mediate the hypothermic response of MTII. This effect was also observed using others melanocortin agonists [266]. However, MC4R seems to be essential for the induction of the thermogenic and cardiovascular effects of melanocortin system since the hyperthermic effect of MTII was lost in the MC4R knockout mice [261,264,267]. In summary, these data indicate that complex interactions in the ARC regulate leptin actions on thermogenesis, notably mediated by the melanocortin system. However, as demonstrated with the use of genetically modified models, other signalling pathways may be implicated in this regulation.

### 6.7. Leptin Actions in Extra-Hypothalamic Areas

Leptin also acts directly on other extra-hypothalamic areas, such as the NTS [230]. The neurons of the NTS receive inputs from vagal afferences and project locally within the brainstem and hypothalamic areas involved in the sympathetic regulation of BAT [268]. As NTS neurons express LepR, NTS specific leptin administration leads to a reduction of body weight associated with a reduction in food intake [269]. Although, it seems that leptin alone is unable to regulate BAT thermogenesis via the brainstem, a link between leptin and thyroid releasing hormone (TRH) in this region has been postulated to activate BAT thermogenesis [270,271,272,273]. Interestingly, in addition to the ARC, the NTS is the only other central region where POMC neurons are located. The possibility that POMC neurons in the NTS could be implicated in energy balance (as ARC POMC neurons) has been postulated; however, a study led by Huo and colleagues, found that leptin did not stimulate STAT3 phosphorylation or c-Fos expression in this area, concluding that POMC neurons in the ARC and the NTS were differentially regulated by leptin [257].

### 6.8. Browning and Leptin Central Action

Leptin participates in the regulation of the sympathetic tone of BAT and WAT, and its secretion by the adipocytes is directly controlled by the SNS [274,275,276]. The direct action of leptin in the browning process still remains incompletely understood. In the following section, we will review the different leptin-sensitive brain areas involved in this process.

Some evidence supports that central leptin stimulates gonadal WAT browning via the activation of PI3K signalling in the CNS [277]. Recent studies have shown that ICV leptin infusion resulted in a moderate increase in the expression of several browning genes in inguinal fat. Interestingly, the co-infusion of leptin and insulin induced a higher increase in WAT browning, mediated by POMC neurons located in the ARC and involving the PI3K signalling pathway [207]. The combined inactivation of protein tyrosine phosphatase 1B (PTP1B) and T-cell protein tyrosine phosphatase (TCPTP) stimulates leptin and insulin signalling in POMC neurons increasing browning and energy expenditure [207]. Additionally, the deletion of TCPTP in AGRP-expressing neurons in murine models had the same effects—increasing energy expenditure and browning, resulting in weight loss [278].

Browning is a dynamic process that is specific to different fat depots. O-linked β-N-acetylglucosamine (O-GlcNAc) is an intracellular carbohydrate implicated in different cellular processes, and its specific genetic ablation in AgRP neurons (via inhibition of neuronal excitability) promoted WAT browning. In contrast to this, the acute activation of AgRP neurons suppresses browning preferentially in retroperitoneal and inguinal WAT [279]. As mentioned above, melanocortin neurons are important targets for leptin-induced thermogenesis and accordingly, the deletion of MC4R in sympathetic preganglionic neurons revealed that they are essential factors not only for diet- and cold-induced thermogenesis but also for browning of inguinal WAT [280].

Forkhead box C2 protein (Foxc2), a transcription factor expressed in cardiac cells, mammary gland, liver and adipose tissue, has been shown to: (i) stimulate mitochondrial metabolism, (ii) promote brown adipocytes development [281] and (iii) induce browning in white adipocytes [282,283] by increasing UCP1 mRNA levels in WAT [284]. Recently, Foxc2 and leptin were shown to be functionally associated with the browning process. Foxc2 promotes the browning of WAT through LepR signalling; specifically, cAMP Response Element Binding protein (CREB), a positive transcriptional regulator of leptin, binds the leptin promoter region to potentiate the effects of Foxc2 on WAT browning through the JAK2/STAT3 signalling pathway that is necessary for the activation of the process [284].

Leptin plays a critical role in adipogenesis by regulating the Hedgehog (Hh) signalling pathway. Recently, it has been reported that leptin inhibited Hh signalling, promoting white adipocyte browning and decreasing the adipose weight of HFD-induced obese mice [285]. Since beige adipocytes have been recently identified, more studies linking leptin and browning are necessary to determine the exact role of leptin in this process.

### 6.9. Leptin Actions at Peripheral Level

Although central actions of leptin have been shown to play an important role in the regulation of energy expenditure, the existence of other LepR isoforms expressed widely in the periphery [108,124,286,287] suggests that leptin effects could also be mediated by peripheral tissues.

It has been demonstrated that leptin could have auto- and paracrine effects on adipocytes, potentially contributing to the weight- and fat-reducing activity of leptin [288]. Other authors have evaluated whether the leptin could modulate the cardiovascular system through a peripheral action, but seemingly the main effect remains mediated by the DMH LepR-expressing neurons [289]. Interestingly, LepRb expressing skeletal muscle is another tissue implicated in thermogenesis [108]. Dulloo and coworkers showed, that leptin could have direct thermogenic effects in skeletal muscle. By measuring oxygen consumption in ex vivo muscle samples, they observed that leptin also stimulated thermogenesis by a direct leptin-LepR interaction [290]. Although some studies have demonstrated that leptin could play a role at a peripheral level as stated by LepR expression in peripheral tissues, the main action of leptin seems to be mediated at a central level.

## 7. Leptin Polymorphism and Metabolic Implications

Several attempts have been made to show a possible association between leptin and LepR polymorphisms and metabolic disturbances leading to obesity. Specifically, the LEP A19G mutation in *Lepob* and LEPR Q223R, K109R and K656N mutations in *LepR* have been proposed to be associated with obesity; however, the first results obtained from a meta-analysis of published studies were not able to establish a strong link between leptin and LepR polymorphisms and obesity [291]. Interestingly, it has been found that some people with specific mutations in *LepR* (*LEPR 109KK*) tend to prefer sweet food, implying the need of a personalized medical intervention when treating obesity [292].

Additionally, single nucleotide polymorphisms (SNPs) that could influence metabolic features have been explored in several studies [293,294]. For example, research conducted on people in some specific Asia-Pacific regions, concluded that the Q223R *LepR* SNP could be associated with obesity [295,296,297,298] and type 2 diabetes [299]. Other interesting *LepR* polymorphisms such as K109R and K656N have suggested an association with childhood obesity [297]. These SNPs have also been shown to be strongly associated with obesity in a Chinese population when both were simultaneously present with a LEP 3’flanking region polymorphism [300].

SNP studies in an obesity-driven context have been performed all over the world covering in a variety of ethnic groups, but very little conclusive data have been obtained. While some studies concluded that there was a significant association between polymorphisms and obesity or other metabolic disorders [297], others subsequently failed to find any association [301,302,303]. For a more detailed summation, *Lepob* and *LepR* polymorphisms and their implications in obesity we would refer you to previously published excellent reviews on the subject [291,294]. Although more research needs to be conducted to elucidate the importance of polymorphisms in obesity development and occurrence, it remains an interesting factor to be considered for future anti-obesity therapeutic approaches.

## 8. Leptin as a Therapeutic Approach to Correct Obesity

Following its discovery [103], leptin was immediately described as the anti-obesity miracle cure. Since then, leptin-based therapies have been mainly developed for the treatment of congenital leptin deficiency [129,130], leptin deficiency in lipodystrophy patients [304] and hypothalamic amenorrhea [305,306] leading to the improvement of their phenotypes, including normalization of endocrine axes, decrease in insulin resistance and improvement of lipid profile and hepatic steatosis [307]. Unfortunately, the leptin resistance observed in obese rodents and humans [124,308,309,310] has dismissed the idea of leptin as a possible treatment to treat obesity. In the first clinical trial, a dose-response relationship with weight and fat loss was observed with subcutaneous recombinant leptin injections in both lean and obese subjects, even in those who were hyperleptinemic [311]. However, even with the highest doses of recombinant leptin, huge variability in the magnitude of weight loss among individuals was observed, once again raising the idea of leptin resistance. Other studies which combined leptin therapy with lifestyle management showed similar results in terms of dose response, with observable effects on body weight only with higher doses and after strict dietary interventions [312,313,314,315]. In agreement with these findings, no additional reduction of body weight was observed in overweight/obese hypoleptinemic patients after Roux-en-Y gastric bypass augmented with leptin therapy [316]. Of note, the increased circulating leptin levels after chronic therapy induces the production of anti-leptin antibodies in caloric restricted obese subjects [317].

Interestingly, recent preclinical evidence has suggested that leptin resistance associated with obesity could be overcome which course with compensatory hyperleptinaemia [318,319]. This causes reduced efficacy of leptin replacement therapies in obese patients. The concept of leptin resistance during obesity could be due to several molecular mechanisms. Firstly, leptin could induce the expression of SOCS3, which blocks the intracellular pathway downstream of leptin receptor signalling [320]. Furthermore, hypothalamic endoplasmic reticulum (ER) stress has been proposed as an important harmful mechanism for central leptin resistance [321]. On the other hand, some evidence support the idea of a central resistance to leptin during obesity which is associated to peripheral hyperleptinaemia, probably more attributable to a reduction in leptin transport across the blood-brain-barrier (BBB) than to hypothalamic leptin insensitivity which would decrease the available amount of leptin at a hypothalamic level [142,322]. However, leptin transport efficiency was not restored after caloric restriction despite hypoleptinaemia [323].

Therefore, prevention and reversion of leptin resistance present a great challenge for researchers and clinicians in the field, as well as the generation of animal models that could be extrapolated to human patients. In this regard, combinatorial therapies with different hormones regulating energy homeostasis are now being developed. Based on these developments, and as some pharmacological treatments are known to increase leptin responsiveness [318], research studies are now focusing on the development of new integrated pharmacological approaches to take advantage of hormonal and molecular synergisms to induce body weight loss. Some interesting findings have already been observed in leptin resistant diet-induced obese rats in which the co-treatment with amylin and leptin (described to have synergistic effects) induced weight loss in a fat-specific way. Of note, hypothalamic leptin signalling was partially restored with an up-regulation of basal and leptin-stimulated signalling in the hindbrain area postrema. In the same study, a similar effect was observed in obese and overweight subjects: the co-administration of recombinant human leptin and the amylin analog pramlintide elicited weight loss of a higher magnitude than both treatments alone [324]. In line with these results, obese and overweight patients subjected to hypocaloric diet and pramlintide treatment for 4 weeks followed by co-administration of metreleptin (a leptin analog) for 20 weeks showed sustained lower weight than patients treated with a monotherapy [325]. Despite these promising results, the clinical trial was halted due to safety concerns.

Remarkably, based on the finding that cholecystokinin (CCK) synergizes with amylin to inhibit food intake in lean mice [326], a triple combination of amylin/leptin/CCK was tested in diet-induced obese rats, resulting in increased body weight loss, reduction of food intake and adiposity than amylin/leptin administration alone [327].

This synergistic effect of leptin is not exclusive to amylin and/or CKK. In recent years, novel factors influencing leptin sensitivity have been explored in several animal studies. Interestingly, some studies have found that GLP-1 receptor agonists co-administered with leptin reduced food intake and body weight in a magnitude that none of the treatments separately could induce [328,329,330,331]. In this regard, when co-administered with leptin, liraglutide (a GLP-1 receptor agonist approved for the treatment of type 2 diabetes) presumably enhanced pSTAT3 after the inhibition of PTP1B [331]. Thus, it was found that the co-administration of high-potency leptin analogs with exedin-4 (glucagon-like peptide 1 receptor agonist) or fibroblast growth factor 21 (FGF21) in diet-induced obese mice (after a change to chow diet and 30% body weight loss) induced restoration of leptin responsiveness. Remarkably this effect was due to the drug co-administration, since leptin alone in animals with similar body weight loss was unable to recapitulate the observed effects [332]. However, these results were based on a change of diet, from HFD to chow diet, and taking this into account, the same group used a different approach in which the reduction in body weight was induced by the administration of a co-agonist targeting glucagon receptor and GLP-1 receptor followed by the administration of high-potency leptin analogs. This induced a greater reduction in body weight, food intake and improved glucose and lipid metabolism than the animals treated with GLP-1/glucagon molecule alone [333].

Research in this field is continuously developing new leptin sensitizers including: meta-chlorophenylpiperazine (an activator of (5-HT) 2c receptors) [334], oxytocin [335] or more recently, uroguanylin [336]. Although results have been promising, its appropriateness in terms of effectiveness and safety in humans remain to be proven. Nevertheless, it opens a promising door for the future development of an effective therapy against obesity.

## 9. Conclusions

The pressing need of effective anti-obesity treatments is driving research to identify new therapeutic targets. Since the discovery of BAT in adult humans [41,60,61,62], a great effort has been made to unravel the activators and molecular pathways leading to BAT activation. BAT is an important organ in the maintenance of body temperature through dissipation of heat by mitochondrial uncoupling, namely thermogenesis [17], and it has the potential capacity to reduce fat mass and regulate glycemic and lipid metabolism [73,74,75,76,77,78,79,80,83,84,85]. Several attempts have been made to target BAT activation in humans, with by far the most potent being cold-induced thermogenesis. However, the high variability of results necessitates an effective pharmacological intervention to satisfactorily activate BAT [62,65]. Due to its anti-obesogenic effect affecting both food intake and energy expenditure, leptin is one of the principal druggable targets to fight obesity and its comorbidities [129,130,318]. While leptin levels are correlated with adipose tissue mass, crucially many other factors can modulate its expression and secretion, as well as its interaction with LepR [110,111,337,338]. Leptin stimulates BAT thermogenesis through central LepRs acting mainly through SNS [188,189,190]. Several hypothalamic areas, such as the DMH, POA, PVH, VMH and ARC, as well as extra-hypothalamic areas as the NTS are involved in leptin-induced thermogenesis [172]. The effects mediated by melanocortin system neurons [141,255] and AgRP/NPY neurons [142,145] of the ARC are of great interest. The classical central regulation of BAT function involves SNS modulation, however the molecular details of that interaction remain elusive. In this regard, recent findings have challenged this view and are still under discussion [56,57,58]. It is worth noting that several lines of evidence support that the leptin/LepR–SNS axis plays a major role in the regulation of energy metabolism, however recent studies were not able to observe any thermogenic response to leptin [193,194,195,196,197,198,199], supporting the idea that the exact role of leptin on thermogenesis still remains unclear. Currently, leptin replacement therapy is being used successfully for the treatment of congenital leptin deficiency, leptin deficiency in lipodystrophy patients and hypothalamic amenorrhea only [129,130,304,305,306]. Attempts to use leptin by itself as an obesity treatment have been disregarded on account of the leptin resistance found in obese patients. Several approaches of combined therapy of leptin with other anti-obesogenic molecules are being tested in preclinical studies, such as amylin, CKK, GLP1, FGF21 and insulin, among others [324,325,326,327,328,329,331,332,333,339]. Although some promising results have been obtained, additional work will be necessary to validate if our current belief in the anti-obesogenic effect of thermogenesis will be clinically relevant for the treatment of obesity.
